# Supplementary material for: Parallel evolution of dominant pistil-side self-incompatibility suppressors in Arabidopsis
Source: Nat Commun. 2020 Mar 16;11:1404. doi: 10.1038/s41467-020-15212-0 (PMC7075917; doi:10.1038/s41467-020-15212-0)
Supplement: Supplementary file 1 — Supplementary Information [file 41467_2020_15212_MOESM1_ESM.pdf]

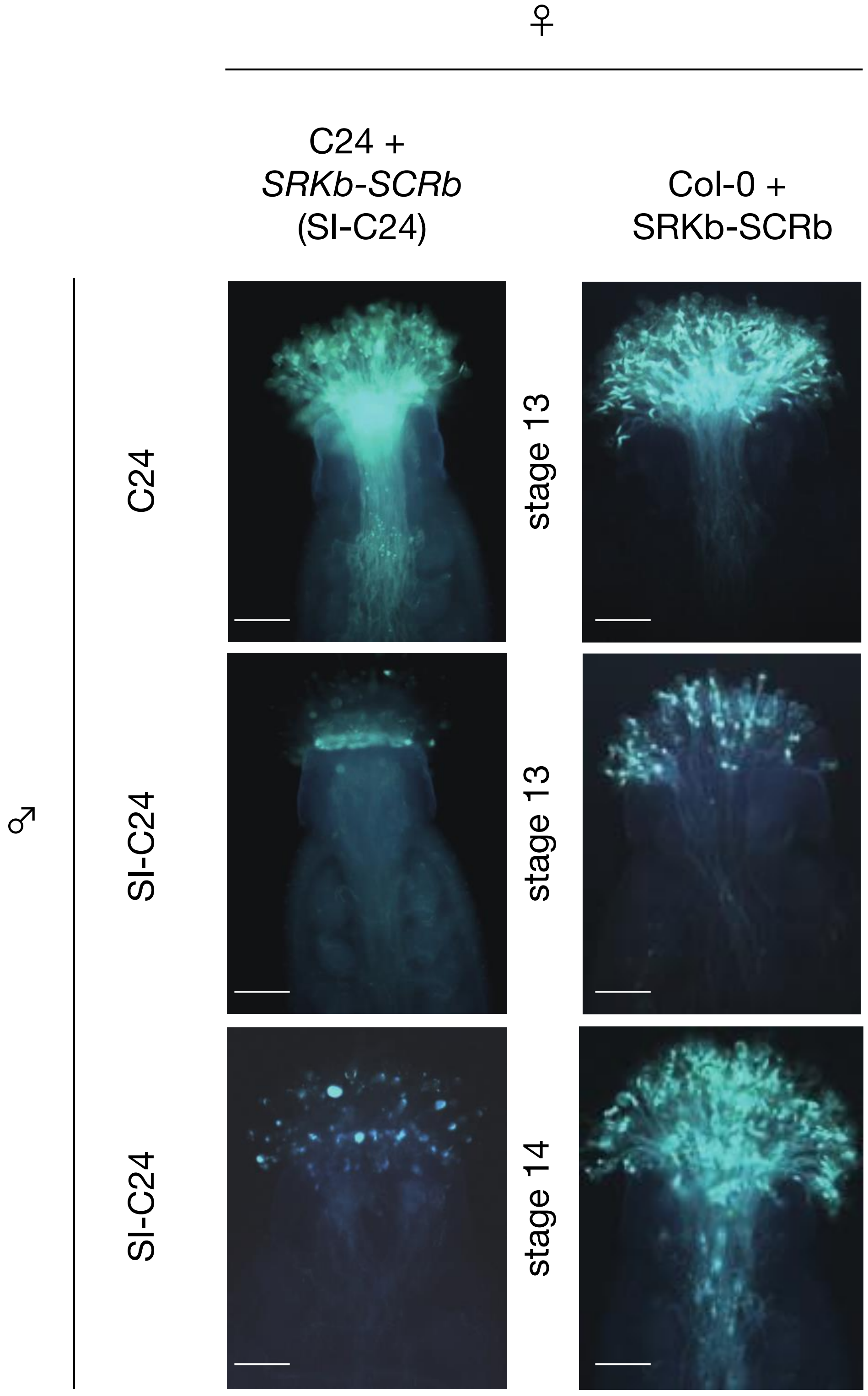

Supplementary Figure 1 Transient SI expression in the Col-0 strain
Images of aniline blue-stained pistils three hours after pollination. Pistils at flowering stages 13 and 14 were pollinated with the pollen from the indicated strains. Scale bars = 100 µm.

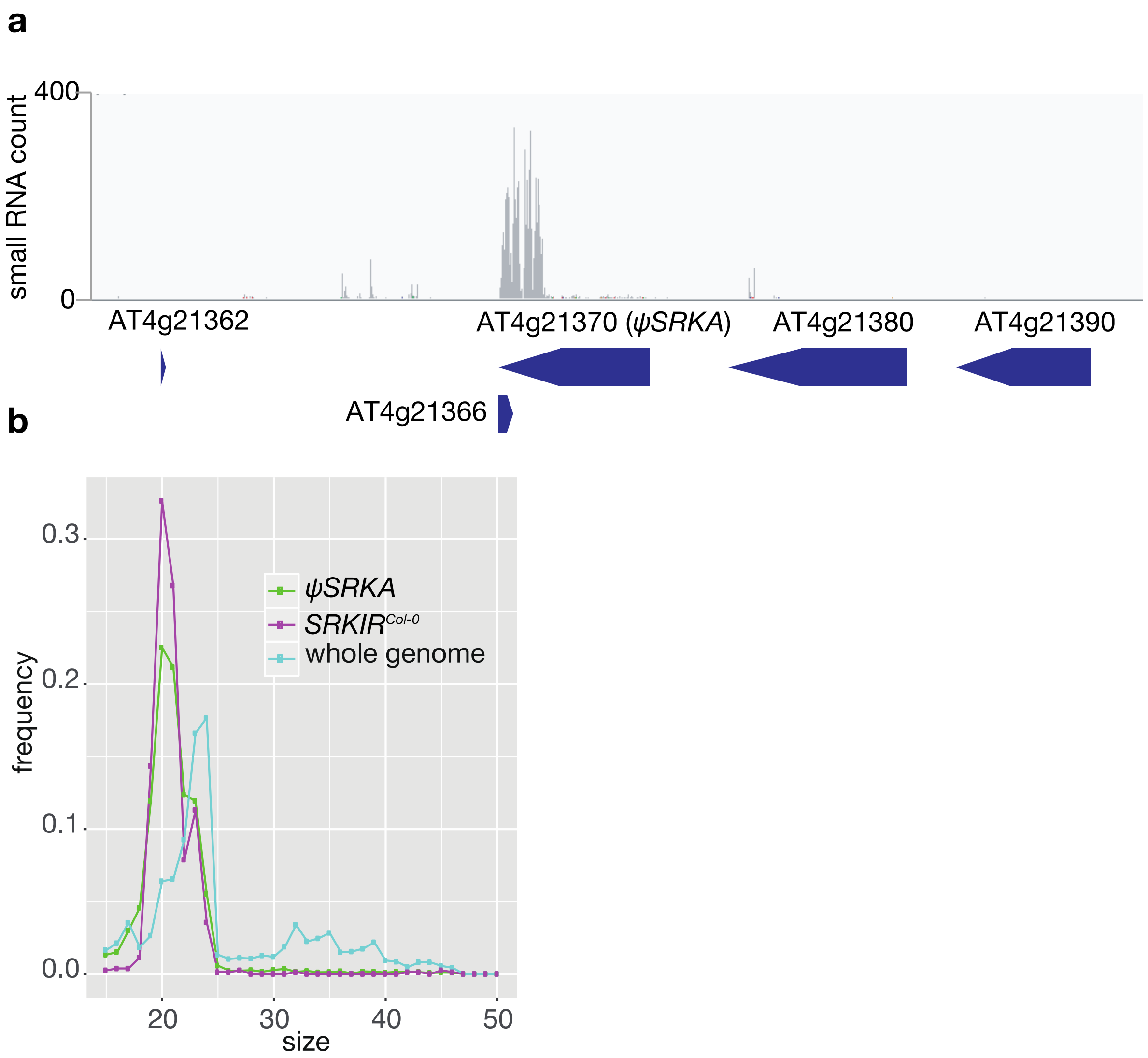

Supplementary Figure 2. Accumulation of small RNAs in Col-0 stigmas that map to the pseudogenized *S* locus

a) Visualization of small RNAs from stigmas that map to the region spanning the pseudogenized *S* locus in Col-0 by IGV. **b)** A line graph showing the distribution of sizes of the small RNAs isolated from Col-0 stigmas. Size profiles of the reads that mapped to the *ψSRKA* locus (*ψSRKA*), the *SRKIR*$_{Col\text{-}0}$ region only (*SRKIR*$_{Col\text{-}0}$) or the whole genome are shown.

```
SRKIR^Col-0   102  cggctacatgtctcccgaatacgcaatggaggggatattctcggtaaaatc  152
Native SRKb   2079 cggttacatgtctccagaatacgcgatggatgggatattctcggtaaaatc  2129
synSRKb       2079 cggatatatgagtcctgagtatgctatggatggaattttttctgttaagtc  2129

SRKIR^Col-0   153  ggatgttttcagcttcggggttttggttcttgagattgtcagtggaaagag  203
Native SRKb   2130 ggatgttttcagctttggggtcttggttcttgagattgtcagtggcaaaaa  2180
synSRKb       2130 tgatgtctttagtttcggagttttagtacttgaaatagtttctggtaaaaa  2180

SRKIR^Col-0   204  gaacagaggattccacaactccggccaagacaataatcttctcggctacgt  254
Native SRKb   2181 gaacaggggattctacaactcaaaccaagacaataacctgctcggctacg-  2230
synSRKb       2181 aaatagaggtttttataattctaatcaggataacaatcttcttggttatg-  2230

SRKIR^Col-0   255  aagcttaaaccgcttaagttttttattttgtctcgttcgattgataacaca  305
Native SRKb        ---------------------------------------------------
synSRKb            ---------------------------------------------------

SRKIR^Col-0   306  attttttttatctttattaacagacgtgggagaattggaaagaaggaaaag  356
Native SRKb   2231 ------------------------cgtggagaaactggaaggaaggaaaag  2257
synSRKb       2231 ------------------------cttggaggaactggaaggagggtaagg  2257

SRKIR^Col-0   357  ggctagagatcgtcgattcgataattgtagattcctcatcatcaatgtcat  407
Native SRKb   2258 gcctagaaatcctagatccattcatcgtagattcatcatcatctccttcag  2308
synSRKb       2258 gacttgagattcttgaccctttttattgttgacagttctagtagtccatctg  2308

SRKIR^Col-0   408  tgttccaaccacatgaagtcttaagatgcatacagattggtcttttatgtg  458
Native SRKb   2309 cgtttcgaccacatgaagtcctaagatgcatacaaattggtctcttgtgtg  2359
synSRKb       2309 ctttccgtcctcacgaggttttaaggtgtattcagatcggattgctttgcg  2359

SRKIR^Col-0   459  ttcaagaacgtgcagaggacagaccaaagatgtcgtcggtgatt         502
Native SRKb   2360 ttcaagaacgtgccgaggatagaccagtgatgtcgtctgtagtg         2403
synSRKb       2360 tgcaggagcgagctgaagacaggccggttatgagcagcgttgtt         2403
```

Supplementary Figure 3. Sequence alignment of *SRKIR*$_{Col\text{-}0}$ (AT4G21370), native *SRKb* (AB052756), and *synSRKb*

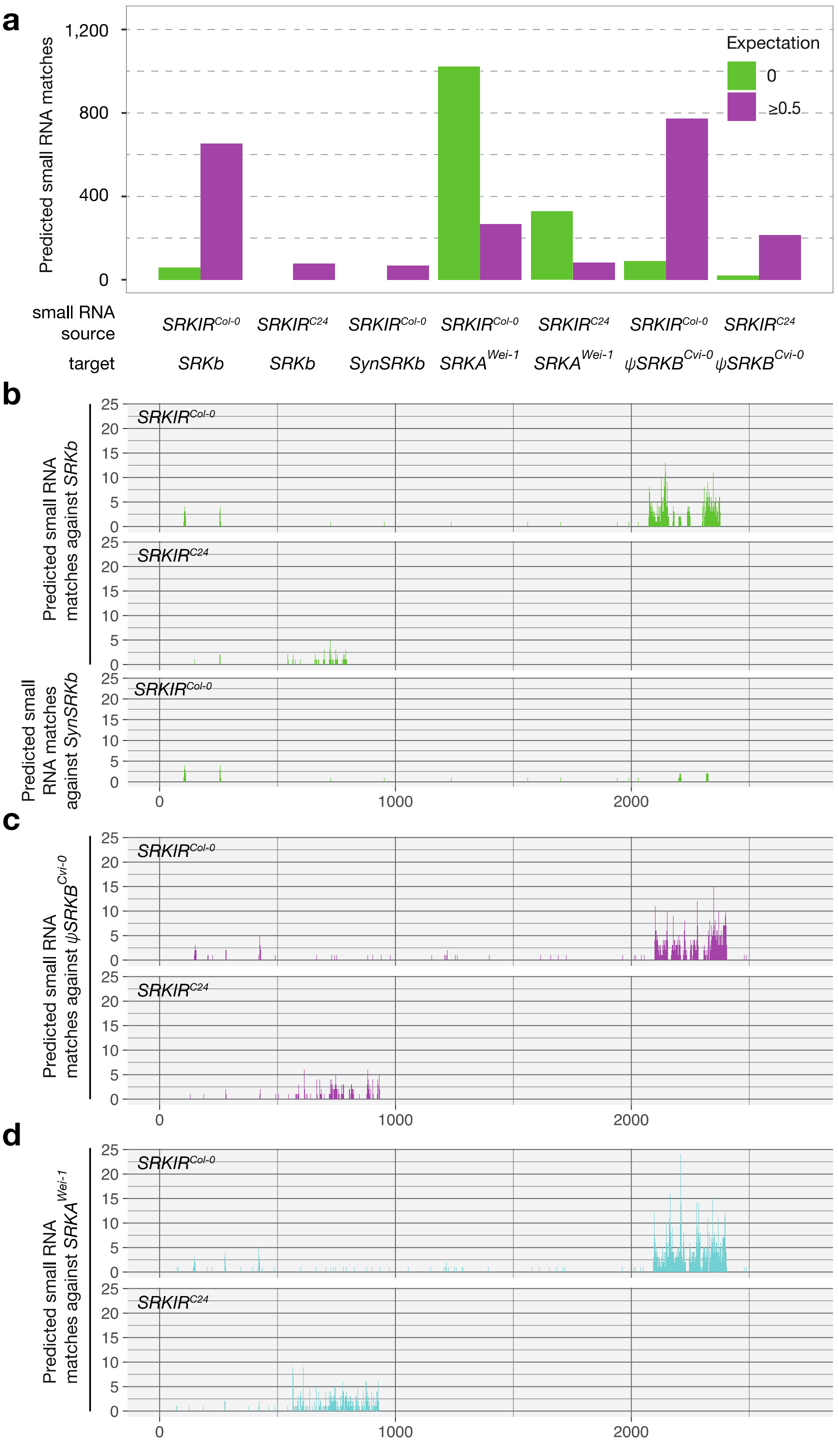

Supplementary Figure 4. a) The numbers of predicted small RNA matches between different combinations of potential small RNA sources (*SRKIR*$_{Col\text{-}0}$ or *SRKIR*$_{C24}$) and different *SRK* targets. From the small RNAs mapped to *SRKIR*$_{Col\text{-}0}$ or *SRKIR*$_{C24}$, non-redundant reads sized from 19 to 24 nucleotides (typical for those functioning in transcriptional gene silencing) were used for the analysis. Matches were sorted into two categories: expectation scores = 0 (a perfect match) and expectation scores = 0.5-3.0 (potential matches including mismatches). **b)** Location of the predicted matches within the *SRKb* or *SynSRKb* sequences. **c)** Location of the predicted matches within *ψSRKB*$_{Cvi\text{-}0}$. **d)** Location of the predicted matches within *SRKA*$_{Wei\text{-}1}$. **b)**, **c)** and **d)** All matches with expectation scores of 0-3.0 are displayed.

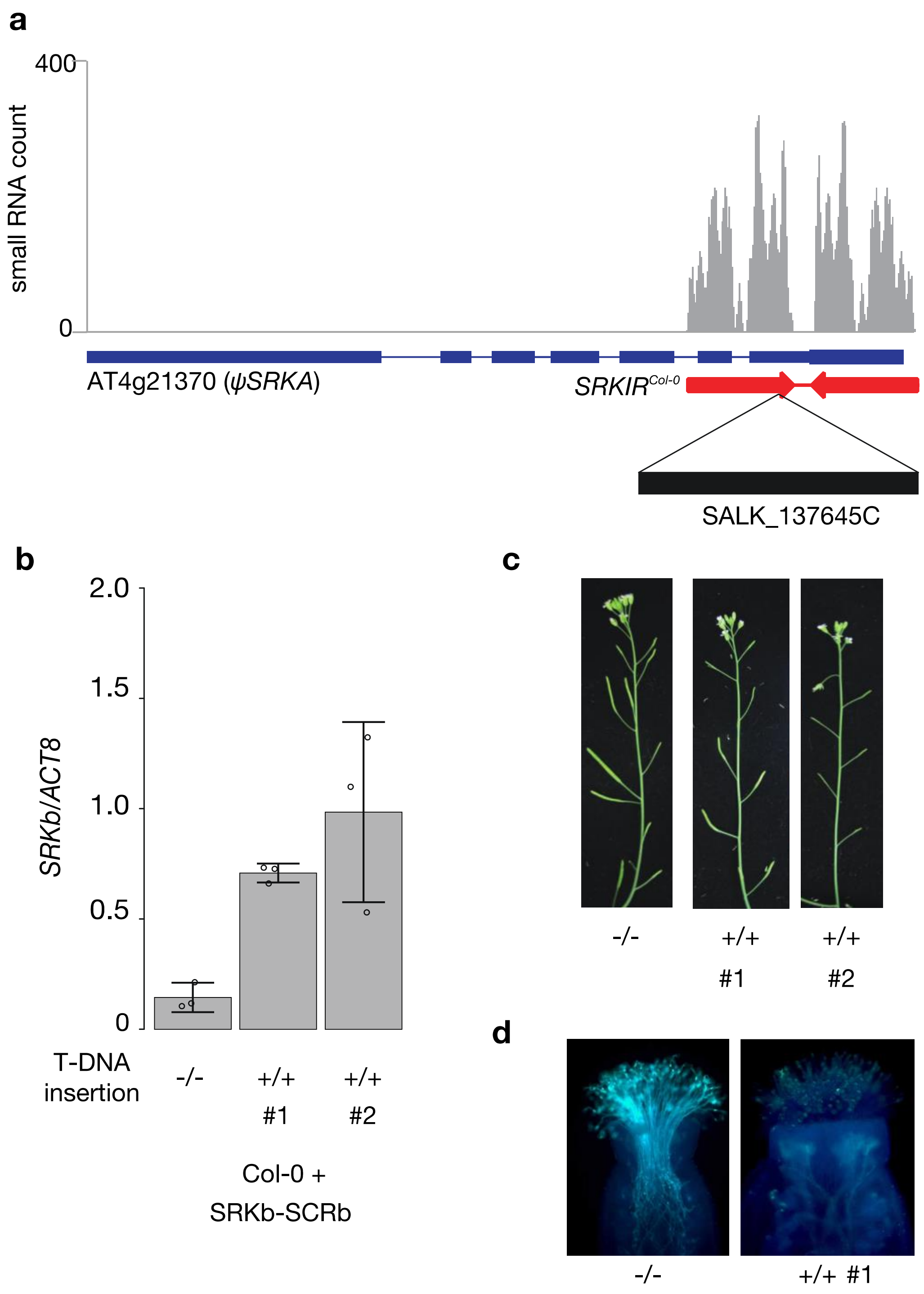

Supplementary Figure 5. a) Closeup view of the *ψSRKA* locus showing exon-intron structures. The approximate location of the T-DNA insertion in SALK_137645C is shown. **b)** Relative mRNA accumulation levels of *SRKb* compared to *ACT8* in stigmatic tissues of the indicated lines. Effect of the homozygous T-DNA insertion (+/+) on *SRKb* mRNA accumulation in the Col-0 background was compared with the no insertion (-/-) line. Two individuals (#1 and #2) were tested. Error bars indicate standard deviations from independent RNA extraction trials (n = 3). **c)** Seed-set phenotype of the two individuals of the Col-0 + *SRKb-SCRb* line carrying homozygous T-DNA insertions within *ψSRKA* (-/-).

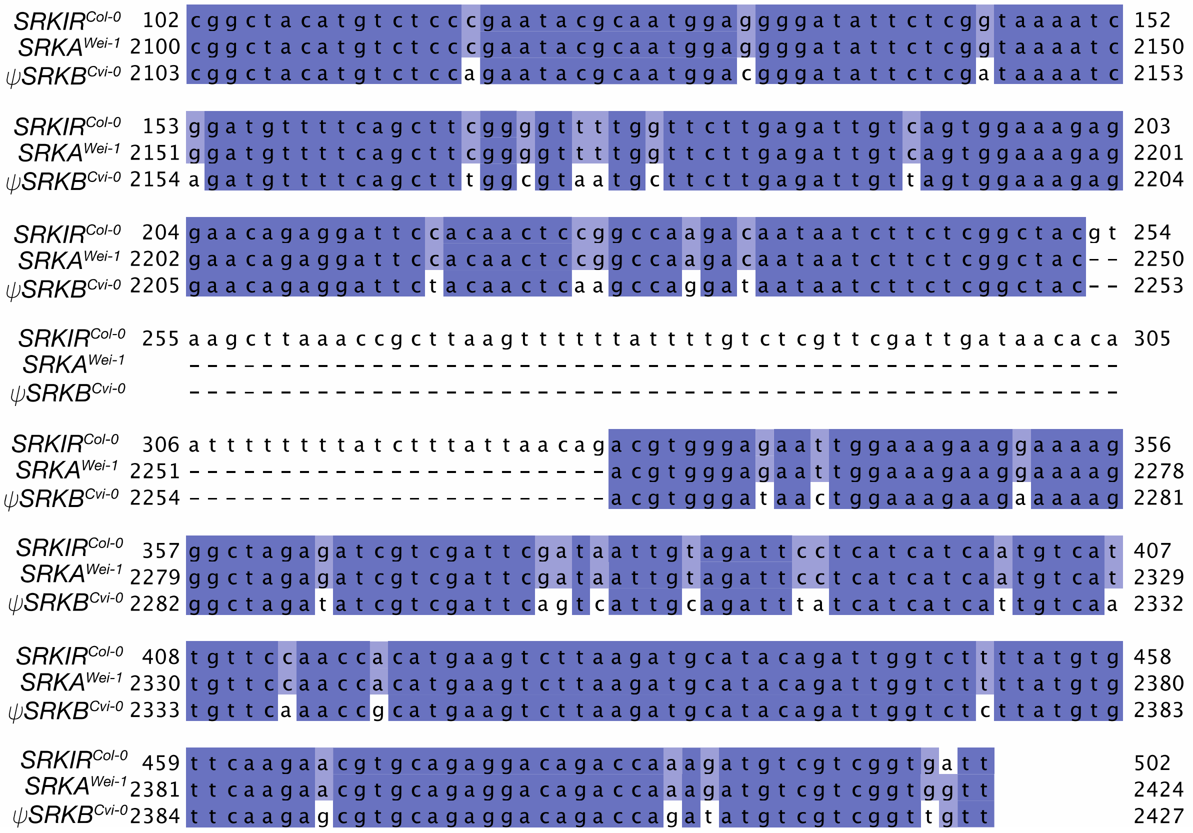

Supplementary Figure 6. Sequence alignment of *SRKIR*$_{Col\text{-}0}$ (AT4G21370) with *SRK* sequences from the Wei-1 (GU723787) and Cvi-0 (AY772644) strains

a

```
1    atgagagtggtggagagagatcgaaagatgagaggtgaattaccaaacaa   50
51   acaccattcttacaccttcttcgttttcctttttctttcttctttaattct  100
101  ggttccctgatctctctatctctgtcaatactttgtcagctacagaatcc  150
151  ctgacaatctcaagcaacaaaaccattgtgtctcctggtggtgtcttcga  200
201  gcttggtttcttcagaatccttggggatagttggtaccttgggatttggt  250
251  acaagaaaatctctcagagaacctatgtatgggttgccaatagagacact  300
301  cctctctctaatcctattggaatcctcaaaatctccaatgctaaccttgt  350
351  catcctcgataattctgatacgcatgtttggtcaacgaatctgactggtg  400
401  cggtgagatcttctgtggtggcagagcttcttgacaatggcaattttgtt  450
451  ctaaggggctccaaaatcaatgaatcagatgagtttttgtggcagagttt  500
501  cgatttcccgacagatactttattaccgcagatgaaattgggTCGGGATC  550
551  ACAAAAGAGGGCTTTAACAGATTCGTCACATCCTGGAAAAGCTCATTTGA  600
601  TCCGTCAAGCGGGAGTTTCATGTTCAAACTCGAAACTCTAGGATTACCTG  650
651  AGTTTTTTGGTTTTACCAGCTTTTTGGAAGTGTACCGGAGCGGCCCTTGG  700
701  GATGGACTCCGGTTTAGCGGCATTCTGGAGATGCAACAATGGGACGATAT  750
751  TATCTACAACTTCACGGAGAATAGAGAGGAGGTCGCTTACACTTTCCGAG  800
801  TTACCGACCACAACTCCTACTCAAGATTGACAATAAATACCGTAGGACGT  850
851  TTAGAGGGATTCATGTGGGAACCAACACAGCAAGAGTGGAACATGTTCTG  900
901  GTTTATGCCAAAGGACACTTGTGATCACAAGTGTAAGGACCACAAATTCC  950
951  ATACTGATCACAAGTGTAAGGACCACnnnnnnnnnnnnnnnnnnnnnnnn  1000
1001 nnnnnnnnnnnnnnnnnnnnnnnnnnnnnnnnnnnnnnnnnnnnnnnnnn  1050
1051 nnnnnnnnnnnnnnnnnnnnnnnnnnaaagcagaggtacaatgaccagtc  1100
1101 tgcgtgattagtatggaattaGTGGTCCTTACACTTGTGATCAGTATGGA  1150
1151 ATTTGTGGTCCTTACACTTGTGATCACAAGTGTCCTTTGGCATAAACCAG  1200
1201 AACATGTTCCACTCTTGCTGTGTTGGTTCCCACATGAATCCCTCTAAACG  1250
1251 TCCTACGGTATTTATTGTCAATCTTGAGTAGGAGTTGTGGTCGGTAACTC  1300
1301 GGAAAGTGTAAGCGACCTCCTCTCTATTCTCCGTGAAGTTGTAGATAATA  1350
1351 TCGTCCCATTGTTGCATCTCCAGAATGCCGCTAAACCGGAGTCCATCCCA  1400
1401 AGGGCCGCTCCGGTACACTTCCAAAAAGCTGGTAAAACCAAAAAACTCAG  1450
1451 GTAATCCTAGAGTTTCGAGTTTGAACATGAAACTCCCGCTTGACGGATCA  1500
1501 AATGAGCTTTTCCAGGATGTGACGAATCTGTTAAAGCCCTCTTTTGTGAT  1550
1551 CCCGA                                               1555
```

b

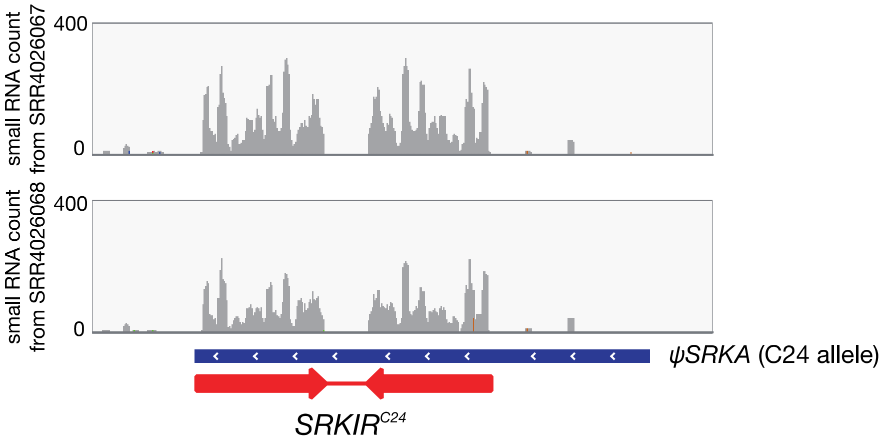

Supplementary Figure 7. a) Pseudogenized *SRKA* sequence of the C24 strain obtained from EF182720. The inverted repeat region is highlighted. **b)** Small RNAs in flower samples of the C24 strain mapped within *ψSRKA*.

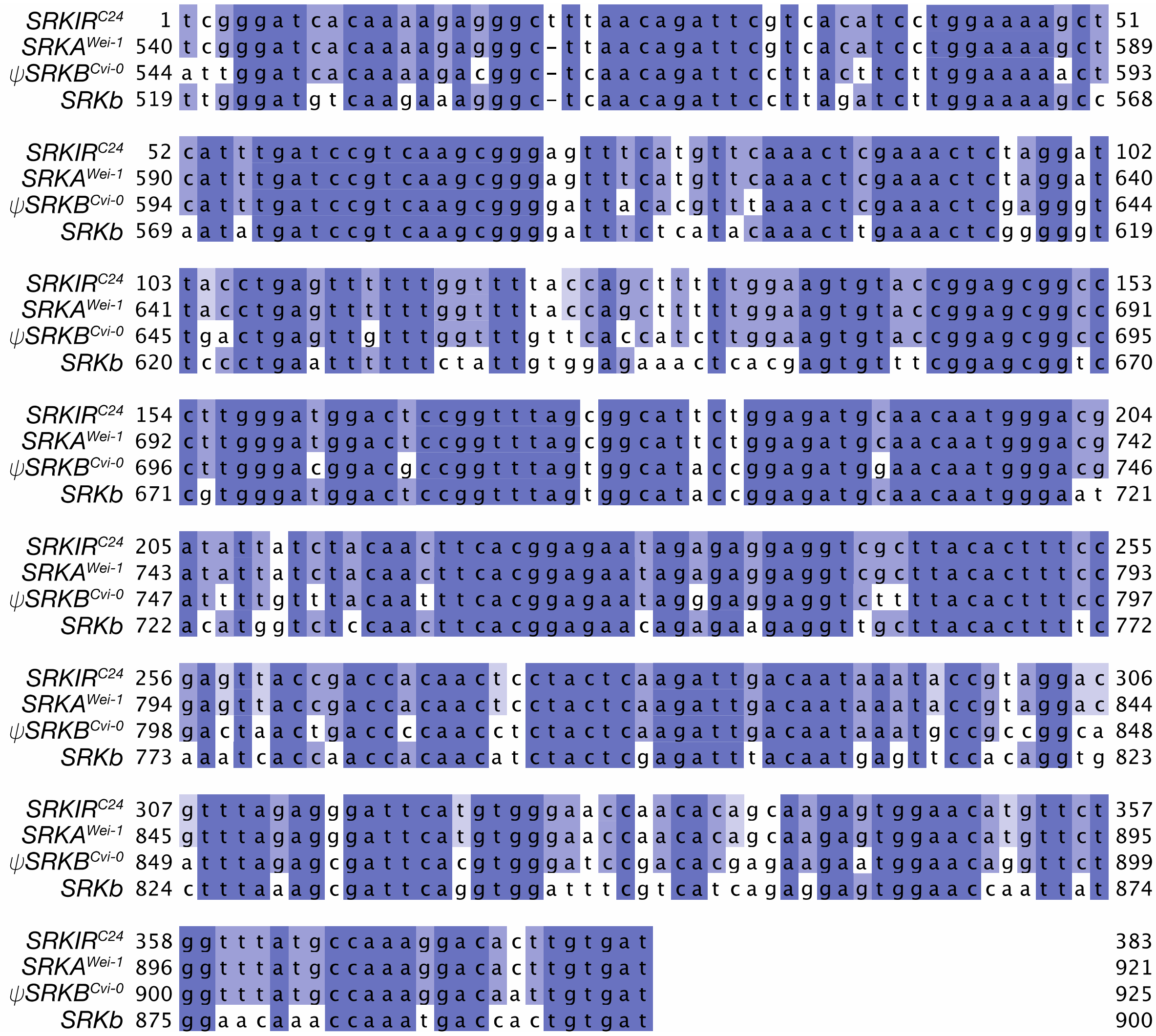

Supplementary Figure 8. Sequence alignment of *SRKIRC24* with *SRKb* (AB052756) and *SRK* sequences from the Wei-1 (GU723787) and Cvi-0 (AY772644) strains

1 a t g a g a g t t g t a g t a c c a a a c t g c c a t c a t t t t a c a t c t t c t t t g t t g t c t t a a t t c t t a t t c g t t c t g 70

71 t a t t t t c c a g c t a c g t c c a t a c t t t g t c g t c g a c a g a a t c a c t g a c a a t c t c a a g c a a a c a a a c c a t t g t 140

141 a t c t c c c g g t g a g g t c t t c g a g c t t g g t t t c t t c a a c c c c g c t g c a a c t t c t c g a g a t g g t g a t c g t t g g 210

211 t a t c t a g g a a t t t g g t t c a a g a c a a a c c t c g a g a g a a c c t a c g t a t g g g t t g c c a a c a g a g a t a a t c c t c 280

281 t c t a c a a t t c c a c t g g a a c t c t t a a g a t t t c c g a t a c t a a t c t c g t c c t t c t c g a t c a a t t t g a t a c c c t 350

351 t g t a t g g t c g a c g a a t c t a a c c g g a g t t t t g c g a t c t c c g g t g g t g g c a g a g c t t c t t t c t a a c g g c a a c 420

421 t t a g t t c t t a a a g a c t c c a a a a c c a a c g a c a a a g a t g g a a t t t t g t g g c a a a g c t t c g a t t a t c c a a c t g 490

491 a t a c t t t a c t t c c a c a g a t g a a a a t g g g t t g g g a t g t c a a g a a a g g g c t c a a c a g a t t c c t t a g a t c t t g 560

561 g a a a a g c c a a t a t g a t c c g t c a a g c g g g g a t t t c t c a t a c a a a c t t g a a a c t c g g g g g t t c c c t g a a t t t 630

631 t t t c t a t t g t g g a g a a a c t c a c g a g t g t t t c g g a g c g g t c c g t g g g a t g g a c t c c g g t t t a g t g g c a t a c 700

701 c g g a g a t g c a a c a a t g g g a a t a c a t g g t c t c c a a c t t c a c g g a g a a c a g a g a a g a g g t t g c t t a c a c t t t 770

771 t c a a a t c a c c a a c c a c a a c a t c t a c t c g a g a t t t a c a a t g a g t t c c a c a g g t g c t t t a a a g c g a t t c a g g 840

841 t g g a t t t c g t c a t c a g a g g a g t g g a a c c a a t t a t g g a a c a a a c c a a a t g a c c a c t g t g a t a t g t a t a a g a 910

911 g g t g t g g g c c t t a t a g t t a c t g t g a t a t g a a c a c g t c a c c g a t t t g t a a c t g t a t t g g a g g g t t t a a g c c 980

981 g a g g a a t c t g c a t g a g t g g a c a t t g a g a a a t g g g t c a a t t g g g t g t g t t a g g a a g a c a c g g c t g a a c t g c 1050

1051 g g t g g a g a t g g g t t t t t g t g c c t g a g a a a g a t g a a g t t g c c a g a t a g t t c g g c g g c a a t t g t a g a t a g g a 1120

1121 c a a t t g a t c t a g g a g a a t g c a a a a a g a g g t g t c t c a a t g a t t g t a a t t g t a c a g c a t a t g c g a g t a c a g a 1190

1191 t a t c c a g a a t g g c g g g t t g g g a t g t g t g a t t t g g a t t g a a g a g c t c t t g g a t a t c c g a a a c t a c g c c a g c 1260

1261 g g c g g c c a a g a t c t t t a t g t t a g a t t g g c t g a t g t t g a t a t t g g a g a t g a a a g a a a c a t a a g a g g a a a a a 1330

1331 t c a t c g g t t t a g c t g t t g g a g c t a g c g t t a t c c t t t t t c t g a g t t c c a t c a t g t t c t g c g t t t g g a g a a g 1400

1401 g a a a c a g a a g c t a c t a a g a g c a a c t g a a g c a c c t a t t g t g t a t c c a a c a a t a a a c c a a g g t t t g c t a a t g 1470

1471 a a t c g c c t g g a a a t a t c a a g c g g g a g a c a c c t a t c t g a a g a t a a c c a a a c g g a g g a t t t g g a a c t t c c a t 1540

1541 t a g t g g a a t t t g a a g c t g t t g t c a t g g c t a c a g a a a a t t t c t c c a a t t c c a a c a a a c t t g g a g a a g g c g g 1610

1611 c t t t g g t g t c g t t t a c a a g g g a a g a t t a c t t g a c g g a c a a g a g a t t g c g g t g a a a a g g c t a t c c a c a a c a 1680

1681 t c a a t t c a a g g g a t a t g t g a g t t c a g a a a c g a g g t g a a g c t g a t c t c g a a a c t t c a g c a c a t c a a c c t t g 1750

1751 t c c g a c t t t t t g g t t g t t g t g t c g a c g a a a a c g a g a a g a t g t t g a t c t a t g a g t a t t t g g a g a a c c t a a g 1820

1821 c c t t g a t t c t c a t c t c t t t a a c a a a a g c c t g a g c t g t a a g c t g a a t t g g c a a a t g a g a t t t g a t a t t a c t 1890

1891 a a t g g t a t t g c t c g g g g a c t t c t a t a t c t t c a c c a a g a t t c a c g g t t c a g g a t c a t t c a c a g a g a t t t g a 1960

1961 a a g c t a g t a a c g t c t t a c t t g a t a a a g a t a t g a c t c c g a a a a t t t c g g a t t t c g g a a t g g c c a g g a t c t t 2030

2031 t g g a c g g g a c g a g a c c g a a g c t a a c a c g a g g a a g g t g g t c g g a a c t t a C G G a T A t A T G a g T C C t G A g T A t 2100

2101 G C t A T G G A T G G a A T t T T t T C t G T t A A g T C t G A T G T c T T t A G t T T c G G a G T t T T a G T a C T T G A a A T a G T t t 2170

2171 c T G G t A A a A A a A A t A G a G G t T T t T A t A A t T C t A A t C A g G A t A A c A A t C T t C T t G G t T A t G C t T G G A G g A A 2240

2241 c T G G A A g G A g G G t A A g G G a C T t G A g A T t C T t G A c C C t T T t A T t G T t G A c a g t T C t a g t a g t C C a T C t G C t 2310

2311 T T c C G t C C t C A c G A g G T t t T A A G g T G t A T t C A g A T c G G a t T g c T t T G c G T g C A g G A g C G a G C t G A a G A c A 2380

2381 G g C C g G T t A T G a g c a g c G T t G T t G T t a t g c t c a g g a g t g a a a c a g a a a c c a t c c c t c a g c c t a a a c c g c c 2450

2451 g g g t t a t t g t g t c g g g a g a a g t c c t t t t g a a a c t g a t t c t t c g a c a c a t g a a c a g c g t g a c g a a t c c t g t 2520

2521 a c g g t t a a c c a a a t c a c c a t c t c g g c c a t c g a c c c t c g g t a a 2562

Supplementary Figure 9. Full sequence of *synSRKb*. Nucleotide bases substituted from the native *SRKb* are indicated in upper cases.

Supplementary Table 1. List of primers used in this study.

Purpose	Pair	Forward primer name	Forward sequence (5' to 3')	Reverse primer name	Reverse sequence (5' to 3')
**Constuction**	**full SRKIRcol-0**	41/If-pCambia-pseudoSRKA-F	GCCAGTGCCAAGCTTCCTTTGATGAGTAGCTCGTC	42/If-pCambia-pseudoSRKA-R	GGGAAATTCGAGCTCCGATGCAAAGAAGACGATGC
	**partial SRKIRcol-0**	41/If-pCambia-pseudoSRKA-F	GCCAGTGCCAAGCTTCCTTTGATGAGTAGCTCGTC	85/Ifl-Cambia-SRKA-R2	GGGAAATTCGAGCTCCTTCTTCTGACGCAATAACCGG
**Cheking the presence of *SRKb* in the segregating population**		SRKbKD_F	caccCAGAAGCTACTAAGAGCAACTGA	NosT-R	TCGCAAGACCGGCAACAGGATTCA
**Checking the presence of T-DNA insertion (SALK_137645C) in the segregating population**	**T-DNA inserted allele**	LBa1	TGGTTCACGTAGTGGGCCATCG	SALK_137645C-RP	TGACAATTGCATTCAAAAGGG
	**Wild-type allele**	Inside 1F	CCGTTTTGTACTCGTATGACG	Col S-H	GCTAACACCAGGAAGGTGGTTGG
**Quantitative real-time RT-PCR**	***SRKb* (both native and Syn)**	SRKb-F5	ACCATCCCTCAGCCTAAACC	SRKb-R5	GGGTTATTGTGTCGGGAGAAG
	SRKA	271/SRK-Wei1-RT-F2	GAAATACCTCAGCCTAAACGG	272/SRK-Wei1-RT-R2	CCGAGCGTTGATGACCGAGA
	pseudoSRKB	305/SRK-Cvi-RT-F1	TCGTCATCGAGTGCACAACGGAACG	306/SRK-Cvi-RT-R1	CTAATCAGCTATTGCTGCTGCTTC
	ACT8	797/act8-F	AGCACTTTCCAGCAGATGTG	798/act8-R	GAAAGAAATGTGATCCCGTCATG
